# A synthetic lethal screen for Snail-induced enzalutamide resistance identifies JAK/STAT signaling as a therapeutic vulnerability in prostate cancer

**DOI:** 10.3389/fmolb.2023.1104505

**Published:** 2023-05-09

**Authors:** Kathryn E. Ware, Beatrice C. Thomas, Pelumi D. Olawuni, Maya U. Sheth, Nathan Hawkey, M. Yeshwanth, Brian C. Miller, Katherine J. Vietor, Mohit Kumar Jolly, So Young Kim, Andrew J. Armstrong, Jason A. Somarelli

**Affiliations:** ^1^ Department of Medicine, Division of Medical Oncology, Duke University Medical Center, Durham, NC, United States; ^2^ Duke Cancer Institute Center for Prostate and Urologic Cancers, Duke University Medical Center, Durham, NC, United States; ^3^ Dr. Kiran C Patel College of Allopathic Medicine, Nova Southeastern University, Fort Lauderdale, FL, United States; ^4^ Centre for BioSystems Science and Engineering, Indian Institute of Science, Bangalore, India; ^5^ Division of Oncology, Lineberger Comprehensive Cancer Center, University of North Carolina at Chapel Hill, Chapel Hill, NC, United States; ^6^ Department of Molecular Genetics and Microbiology, Duke University Medical Center, Durham, NC, United States; ^7^ Department of Pharmacology and Cancer Biology, Duke University, Durham, NC, United States

**Keywords:** high-throughput screens, drug resistance, hormone therapy resistance, epithelial plasticity, collateral sensitivity

## Abstract

Despite substantial improvements in the treatment landscape of prostate cancer, the evolution of hormone therapy-resistant and metastatic prostate cancer remains a major cause of cancer-related death globally. The mainstay of treatment for advanced prostate cancer is targeting of androgen receptor signaling, including androgen deprivation therapy plus second-generation androgen receptor blockade (e.g., enzalutamide, apalutamide, darolutamide), and/or androgen synthesis inhibition (abiraterone). While these agents have significantly prolonged the lives of patients with advanced prostate cancer, is nearly universal. This therapy resistance is mediated by diverse mechanisms, including both androgen receptor-dependent mechanisms, such as androgen receptor mutations, amplifications, alternative splicing, and amplification, as well as non-androgen receptor-mediated mechanisms, such as lineage plasticity toward neuroendocrine-like or epithelial-mesenchymal transition (EMT)-like lineages. Our prior work identified the EMT transcriptional regulator Snail as critical to hormonal therapy resistance and is commonly detected in human metastatic prostate cancer. In the current study, we sought to interrogate the actionable landscape of EMT-mediated hormone therapy resistant prostate cancer to identify synthetic lethality and collateral sensitivity approaches to treating this aggressive, therapy-resistant disease state. Using a combination of high-throughput drug screens and multi-parameter phenotyping by confluence imaging, ATP production, and phenotypic plasticity reporters of EMT, we identified candidate synthetic lethalities to Snail-mediated EMT in prostate cancer. These analyses identified multiple actionable targets, such as XPO1, PI3K/mTOR, aurora kinases, c-MET, polo-like kinases, and JAK/STAT as synthetic lethalities in Snail+ prostate cancer. We validated these targets in a subsequent validation screen in an LNCaP-derived model of resistance to sequential androgen deprivation and enzalutamide. This follow-up screen provided validation of inhibitors of JAK/STAT and PI3K/mTOR as therapeutic vulnerabilities for both Snail+ and enzalutamide-resistant prostate cancer.

## Introduction

The treatment landscape of prostate cancer exemplifies the “two truths” of cancer treatment (Somarelli et al., 2022): While tremendous progress has been made to improve patient outcomes, there also remains an urgent need to overcome the significant challenges imposed by the evolution of treatment resistance and metastasis. From the groundbreaking studies of Huggins and Hodges (Huggins and Hodges, 1972) to the development of novel, second-generation androgen receptor inhibitors (Scher et al., 2012; Beer et al., 2014; Smith et al., 2018; Chi et al., 2019; Fizazi et al., 2019; Armstrong et al., 2022), and anti-androgens (Fizazi et al., 2017; James et al., 2017), much of the existing treatment options for prostate cancer are currently focused on targeting the androgen receptor (AR) signaling axis. These agents have demonstrated significant clinical benefit; however, progression of men treated with these agents in the metastatic, castration-resistant setting is nearly universal.

The evolution of resistance to AR signaling inhibitors is mediated by heterogeneous genetic and non-genetic pathways that include both AR-dependent and AR-independent mechanisms [reviewed in (Blatt and Raj, 2019)]. Among these heterogeneous mechanisms, phenotypic plasticity is a central hallmark of AR signaling inhibitor resistance (Somarelli et al., 2020a). This phenotypic plasticity occurs along multiple, interconnected cellular lineage axes, such as stemness (Alumkal et al., 2020; Rodriguez et al., 2022), epithelial/mesenchymal (Ware et al., 2016; Miao et al., 2017; Bai et al., 2019; Ware et al., 2020), luminal/basal (Stoyanova et al., 2013; Li et al., 2018), and neuroendocrine-like lineages or cell states (Ku et al., 2017; Mu et al., 2017). Phenotypic plasticity along these axes often leads to a loss of AR expression/activity and dependency (Formaggio et al., 2021), as well as additional aggressive features that promote survival and metastasis (Schroeder et al., 2014; Labrecque et al., 2021). New approaches are needed to capitalize on these emerging phenotypic states for therapeutic benefit.

Targeted therapy alters the ecological fitness landscapes of cancer in multiple ways (Somarelli, 2021). The altered fitness landscape of the drugged environment can promote aggressive biology, but can also induce “collateral sensitivities” to novel agents (Acar et al., 2020). This concept, also known as negative cross resistance, has been applied to identify new strategies to treat the evolution of resistance in bacterial infections (Imamovic and Sommer, 2013), malaria (Kirkman et al., 2018), herbicides (Cutti et al., 2021), and pesticides (Wazir and Shad, 2022).

**FIGURE 1:**
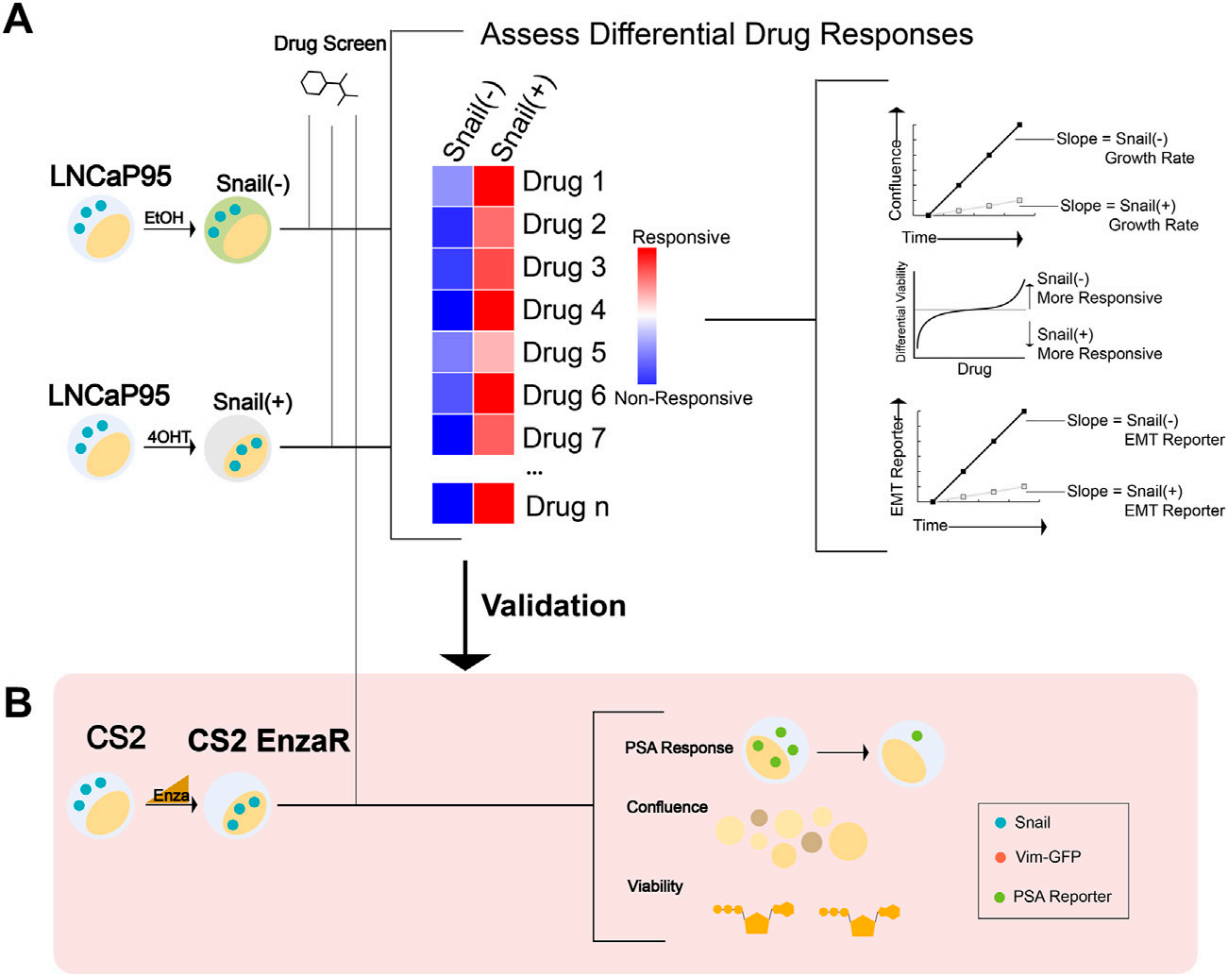
Workflow schematic for synthetic lethal and collateral sensitivity screens. **(A)** A high-throughput screen was performed in LNCaP95-Snail cells to assess differential response across multiple endpoints of confluence, viability (CellTiter Glo), and EMT status via a fluorescence-based reporter. (EtOH = ethanol; 4OHT = 4-Hydroxytamoxifen). **(B)** Screen schematic for a collateral sensitivity screen in enzalutamide-resistant CS2 cells. CS2 EnzaR cells generated by chronic treatment with enzalutamide (ref 16 https://doi.org/10.1101/2020.04.22.050385) were further transduced with a PSA-GFP reporter to assess multiple endpoints including PSA reporter response, confluence, and viability (CellTiter Glo).

In the present study, we combined high-throughput screens with multiparameter endpoint measurements from transcription-based reporters, confluence, and cell viability assays to characterize the therapeutic landscapes of Snail-mediated EMT, enzalutamide resistance, and AR activity (Figure 1A). Our analyses pinpoint histone deacetylases (HDAC), protein kinase A (PKA), PI3K/mTOR, and Janus Kinase (JAK) as key collateral sensitivities to Snail-mediated enzalutamide resistance in prostate cancer cells. Follow-up screens in a model of progressive adaptation to ADT and enzalutamide resistance verified the relevance of these pathways as novel therapeutic vulnerabilities for enzalutamide-resistant prostate cancer (Figure 1B). These analyses provide a deeper understanding of the therapeutic vulnerabilities induced by epithelial plasticity and enzalutamide resistance.

## Materials and methods

### Cell culture models

LNCaP95-Snail (Hu et al., 2012) and CS2 enzalutamide-resistant cells (Ware et al., 2020; Jindal et al., 2023) were cultured in RPMI containing 10% charcoal stripped Fetal Bovine Serum (Sigma, St. Louis, MO) and 1% penicillin/streptomycin (Life Technologies, Carlsbad, CA). CS2 enzalutamide-resistant cell populations were maintained in the presence of 50 μM enzalutamide (Selleckchem, Houston, TX). Cell lines were maintained in standard tissue culture-treated plasticware within a humidified incubator at 37°C and 5% CO_2_. LNCaP95 cells stably expressing inducible Snail were generated as previously described (Ware et al., 2016). Induction of Snail nuclear translocation was mediated by the addition of 4-hydroxy-tamoxifen (4OHT) at a concentration of 20 nM. Ethanol (EtOH) was used as a vehicle control. All cells were authenticated by the Duke DNA Analysis Facility using analysis of short tandem repeats and were verified to be mycoplasma-free.

### Development and testing of MET and PSA reporter lines

We adapted the GIIIcI^2^ MET reporter (Somarelli et al., 2013; Somarelli et al., 2016) for lentiviral transduction by cloning the previously-described vector into the lentiviral vector pLVX-puro using restriction enzymes EcoRI/SmaI (NEB, Ipswich, MA). The PSA reporter was synthesized in the lentiviral expression plasmid, pLV[Exp]-Puro by VectorBuilder (Chicago, IL) to include 2 Kb of the proximal PSA promoter upstream of the enhanced green fluorescent protein (EGFP) open reading frame (Zhang et al., 1996). Cells stably expressing inducible Snail (Addgene plasmid #18798) or indicated reporter plasmids were generated by transduction of LNCaP95 or CS2 cells as described: https://www.addgene.org/protocols/generating-stable-cell-lines/. Confluence and fluorescence were measured with and without EMT induction using Snail activation as described above. For PSA-GFP expressing cells, confluence and fluorescence was quantified with and without AR activation using synthetic androgen R1881(Sigma, St. Louis, MO) at 1 nM.

### High-throughput drug screening

High-throughput screens were performed in collaboration with the Duke Functional Genomics Shared Resource as previously described (Altunel et al., 2020; Somarelli et al., 2020b; Somarelli et al., 2020c; Rao et al., 2020). Briefly, compounds from the Bioactives library (SelleckChem, Houston, TX) were stamped in triplicate into 384-well plates at a final concentration of 1 μM using an Echo Acoustic Dispenser (Labcyte, Indianapolis, IN, United States). Cells and media were subsequently dispensed into plates using a WellMate (Thermo Fisher, Waltham, MA, United States) at a density of 2,000 cells/well for each cell line. Confluence was quantified using an IncuCyte S3 (Gottingen, Germany) live cell imaging system. GIIIcI^2^ and PSA-GFP readouts were quantified by IncuCyte imaging at 24, 48, 72, and 96 h. CellTiter Glo (Promega, Madison, WI) was added at 96 h, and luminescence was read using a Clariostar plate reader (BMG, Berlin, Germany).

### RNA-Seq analysis of EMT scores

Quantification of EMT status for each sample was done using three distinct methods, 76GS, KS, MLR, each of which uses a unique algorithm and gene set. The 76GS scores were calculated based on the expression of 76 genes (Byers et al., 2013). Higher scores correspond to more epithelial states. A 76GS score >0 typically indicates an epithelial phenotype and <0 indicates a mesenchymal phenotype. The score for each sample is computed as the weighted sum of expression values of 76 genes, with the weight factor being the correlation of expression values of that gene with that of CDH1 in the given dataset. KS score was determined based on a Kolmogorov–Smirnov two-samples test (Tan et al., 2014). Using a 218 gene signature, the cumulative distribution functions are estimated for mesenchymal and epithelial signatures, and the maximum difference in cumulative distribution functions is retained as the statistic for the two sample-KS test. KS score ranges from [−1, 1], with negative and positive scores representing mesenchymal and epithelial phenotypes, respectively. MLR scores are provided on a scale of [0, 2]; higher scores are associated with more mesenchymal samples (George et al., 2017). Using an ordinal multinomial logistic regression, the score encompasses an order structure, with a hybrid epithelial/mesenchymal signature situated between the epithelial and mesenchymal phenotypes. Scores are calculated based on the probability assigned for each sample to belong to one of the three phenotypes.

### Data analysis

The primary objective for the high-throughput screen of LNCaP95-Snail cells was to identify synthetic lethality for Snail+ cells. Snail−cells (EtOH-treated vehicle controls) were used as a reference control to calculate differential effects across all parameters. The primary objective for the high-throughput screen of CS2 enzalutamide-resistant cells was to identify collateral sensitivities for enzalutamide-resistant cells. The central hypothesis for this work was that activation of key pathways in Snail+, enzalutamide-resistant prostate cancer can be exploited for therapeutic benefit through synthetic lethal and collateral sensitivity approaches. Experimental data were visualized and analyzed in GraphPad Prism 9 (Boston, MA). Analysis of cell viability by CellTiter Glo was performed by normalizing to the average of all empty (non-drug) wells. Quantification of immunofluorescence images was performed using ImageJ. Briefly, images were converted to 8-bit, adjusted for threshold, and analyzed for particle count and area. For nuclear proportion experiments, nuclear Snail was counted and normalized to total number of cells per field. Imaging of confluence and GFP was compared using repeated measures ANOVA. Linear regression was used to assess correlations between screen analysis parameters, and outliers were considered to fall outside the 95% confidence interval bands. *p*-values < 0.05 were considered statistically reliable.

## Results

### Fluorescence-based reporters enable real-time monitoring of epithelial plasticity

**FIGURE 2:**
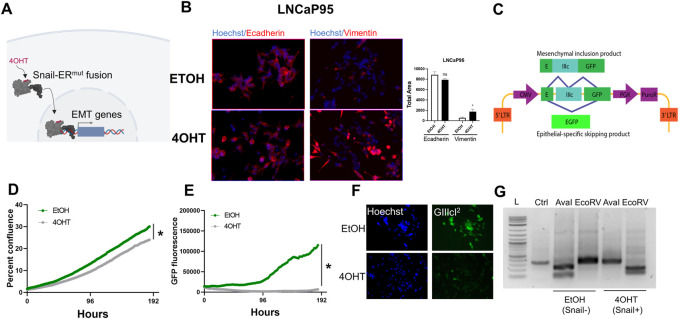
Fluorescence-based reporters to visualize EMT dynamics in a Snail-inducible model. **(A)** Schematic illustration of a Snail-inducible model. **(B)** Immunofluorescence staining of LNCaP95-Snail cells. EtOH serves as a vehicle for Snail induction. 4OHT induces localization of Snail and concomitant downregulation of E-cadherin and upregulation of vimentin. **(C)** Schematic of the GIIIcI^2^ EMT/MET alternative splicing reporter. **(D)** IncuCyte imaging for LNCaP95-Snail confluence and **(E)** EMT induction dynamics (GFP fluorescence). * = *p*< 0.05. **(F)** Fluorescence imaging of LNCaP95-Snail cells treated with EtOH or 4OHT for nuclear staining (Hoechst) and the GIIIcI^2^ EMT/MET reporter (green). **(G)** Endogenous FGFR2 splicing analysis for Snail− and Snail+ LNCaP95 cells. L = 1 Kb ladder, Ctrl = undigested PCR product; AvaI = FGFR2-IIIb-specific restriction digestion; EcoRV = FGFR2-IIIc-specific restriction digestion. All data is representative of a minimum of two independent experiments performed in triplicate.

Prior studies have pinpointed the epithelial plasticity regulator, Snail, as both upregulated during AR inhibition (Miao et al., 2017) and a mediator of enzalutamide resistance through sustained androgen receptor signaling (Ware et al., 2016). LNCaP95 cells are a model of castration-resistant prostate cancer that demonstrate sustained AR expression, cellular plasticity (Hu et al., 2012) and acquire enzalutamide resistance through activation of the transcription factor Snail (Ware et al., 2016). In the present work we sought to develop a system to identify novel synthetic lethality to Snail-induced resistance to enzalutamide. To do this we turned to a Snail inducible LNCaP95 cell line system in which Snail is fused to an estrogen receptor mutant (ER^mut^) whereby 4-hydroxytamoxifen (4OHT) acts as an agonist (Figure 2A). Addition of 4OHT induces estrogen receptor-Snail fusion nuclear localization and activation of Snail (Figure 2A). Addition of 4OHT in the Snail-inducible LNCaP95 prostate cancer cell line leads to cell scattering and upregulation of the mesenchymal marker, vimentin (Figure 2B; Supplementary Figure S1). To track dynamics of Snail-mediated epithelial plasticity we adapted the GIIIcI^2^ fluorescence-based reporter (Somarelli et al., 2013) for lentiviral transduction. The GIIIcI^2^ reporter utilizes the lineage-specific alternative splicing within the ligand binding domain of FGFR2 to control EGFP (Somarelli et al., 2013) expression based on epithelial or mesenchymal phenotype. The EGFP open reading frame is interrupted by the FGFR2-IIIc exon and flanking introns (Figure 2C). Splicing of FGFR2-IIIc in epithelial cells leads to fusion of the EGFP reading frame and subsequent EGFP expression while inclusion of the IIIc exon interrupts the EGFP reading frame and prevents EGFP expression (Figure 2C). Treatment of LNCaP95-Snail cells with 4OHT leads to a modest reduction in confluence, consistent with the known relationship between Snail and cell cycle arrest (Vega et al., 2004) (Figure 2D). Similarly, Snail induction also induces robust inhibition of EGFP expression (Figure 2E) consistent with inclusion of the mesenchymal FGFR2-IIIc exon. A loss of EGFP signal in Snail+ cells is also evident by fluorescence imaging of Snail− (EtOH) and Snail+ (4OHT) cells (Figure 2F). EGFP expression from the GIIIcI^2^ reporter is also consistent with endogenous FGFR2 splicing, in which 4OHT induces a switch from the IIIb to IIIc isoforms, as observed by isoform-specific restriction digestion of FGFR2 RT-PCR products (Figure 2G).

### High-throughput screens identify synthetic lethality to Snail-induced epithelial plasticity

**FIGURE 3:**
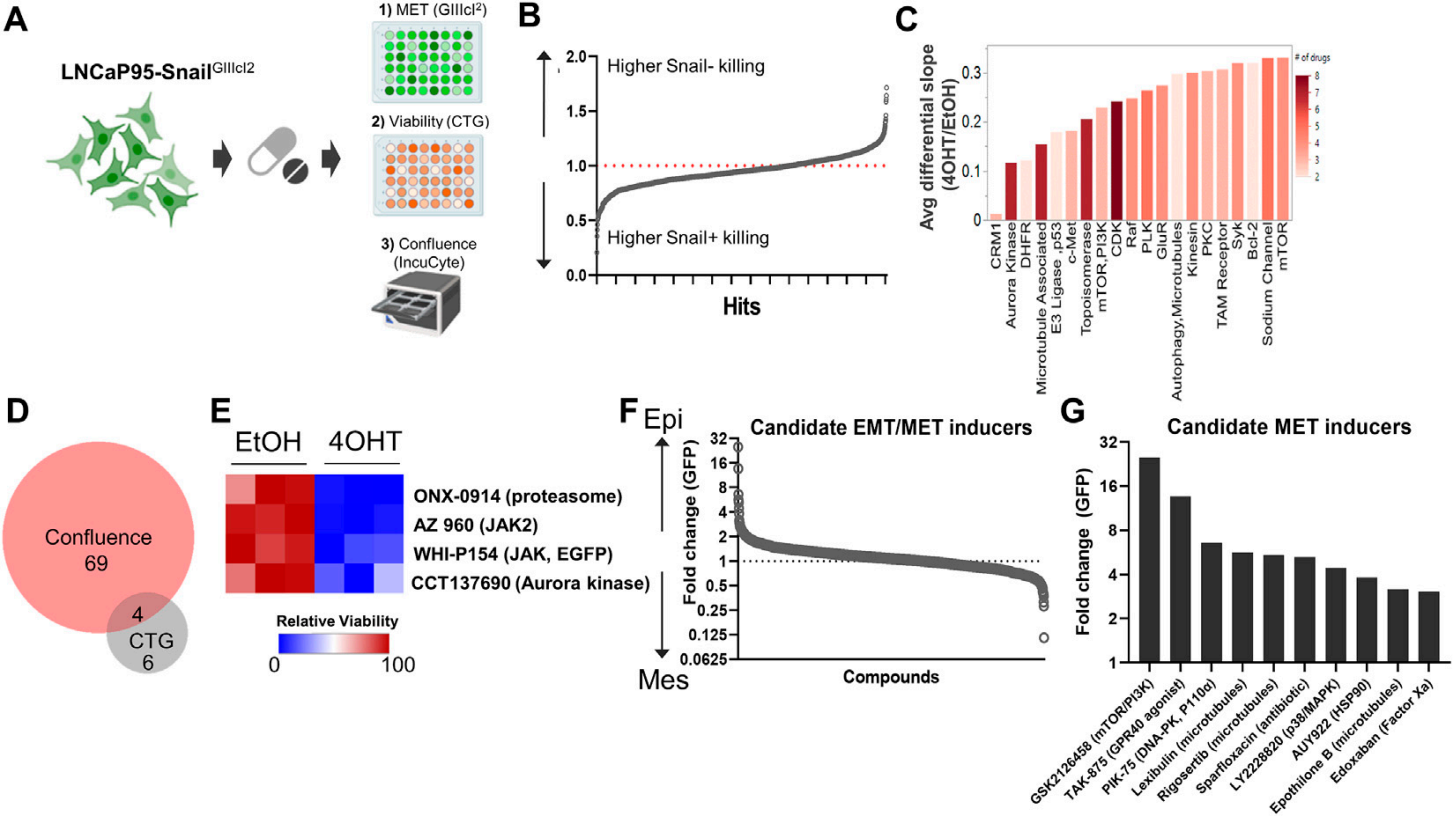
A synthetic lethality screen pinpoints potential therapies for Snail+ prostate cancer. **(A)** Schematic of multi-assay screening strategy. Screens were performed on three replicate plates. **(B)** Top hits with differential response in Snail− and Snail+ cells. Below the 1.0 line indicates drug differentially inhibits Snail+ cells; above the line indicates drug differentially inhibits Snail− cells. **(C)** Top hits grouped by target/pathway ranked by differential slope; color indicates number of drugs per pathway. **(D)** Venn diagram of overlap in compounds that altered both confluence and CellTiter Glo (CTG). **(E)** Overlapping drugs with differential sensitivity in Snail+ cells for both confluence and CTG assays. **(F)** Candidate EMT/MET inducers ranked by GIIIcI^2^ induction (higher GFP = more epithelial; lower GFP = more mesenchymal). **(G)** Top 10 candidate MET inducing compounds, as estimated by EGFP expression from the GIIIcI^2^ reporter.

We applied this Snail-inducible plasticity reporter system to identify compounds with synthetic lethality for Snail+ prostate cancer that could be subsequently validated for activity in models of enzalutamide resistance given the association between Snail expression and enzalutamide resistance (Ware et al., 2016). To do this, we performed a high-throughput small molecule screen using the SelleckChem Bioactives compound library. The Bioactives library contains 2,100 small molecules annotated by target and pathway. The library was designed to include compounds that are structurally diverse, medicinally active, and cell permeable, including both FDA-approved and non-approved compounds (Altunel et al., 2020; Somarelli et al., 2020b; Somarelli et al., 2020c). Screen results were analyzed for cell viability/ATP production by CellTiter Glo at the four-day endpoint, and for cell growth rate and epithelial plasticity status by daily IncuCyte imaging of confluence and GIIIcI^2^ EGFP levels, respectively, for 4 days (Figure 3A; Supplementary Table S1). Analysis of CellTiter Glo values for empty wells revealed a significant reduction in growth for Snail+ cells (Supplementary Figure S2A), which is consistent with the known role of Snail as a mediator of cell cycle arrest. Across the entire compound library 3.8% of compounds inhibited CellTiter Glo signal for Snail− cells by 50% or more, while 22% of the library inhibited Snail+ cells 50% or more (Supplementary Table S1). To identify compounds with differential sensitivity based on Snail expression, we analyzed the differential sensitivity of Snail− and Snail+ cells to all compounds in the library, with a 1.0 representing no difference in sensitivity. Drugs with values <1.0 differentially inhibit CellTiter Glo output of Snail+ cells while drugs with values >1.0 differentially inhibit CellTiter Glo output in Snail− cells (Figure 3B).

In parallel to CellTiter Glo, we also quantified differences in growth rate for all screen compounds with and without Snail induction. Cell confluence was moderately, but significantly, correlated with CellTiter Glo values when comparing all treatment conditions (Supplementary Figure S2B). To identify collateral sensitivities based on growth rate we first calculated differences in slope of the growth rates between Snail− (EtOH) and Snail+ (4OHT) cells. This analysis is shown for a subset of compounds in Supplementary Figure S2C, with compounds in gray having little to no effect on cell growth of Snail− (EtOH) cells and these same compounds inhibiting growth in Snail+ (4OHT) cells. Subsequent annotation by target enabled identification of targets for which >2 drugs hit the same target. Top hits were ranked by their differential slope when comparing Snail+ to Snail−cells. Among these hits were inhibitors targeting signaling molecules and pathways known to be involved in lineage plasticity and prostate cancer therapy resistance, such as aurora kinase, c-MET, and mTOR/PI3K (Figure 3C). Other targets included inhibitors of CRM1 (XPO1), a nuclear shuttling protein, cyclin-dependent kinases, polo-like kinases, and protein kinase C (Figure 3C). To identify synthetic lethality for Snail+ cells, we focused on agents with <50% killing in Snail− (EtOH) cells and >50% killing in Snail+ cells by CellTiter Glo. Among these compounds, comparison of drugs that inhibited both CellTiter Glo production and growth rate by greater than 2-fold in Snail+ cells as compared to Snail− cells revealed four candidate compounds (Figure 3D), including ONX-0914 (immunoproteasome inhibitor), AZ-960 (JAK2 inhibitor), WHI-P154 (JAK3 and EGFR inhibitor), and CCT137690 (aurora kinase inhibitor) (Figure 3E).

We next attempted to identify compounds and pathways that inhibit Snail-induced EMT. To do this we first calculated the fold change in EGFP expression for each compound at day 4 as compared to day 1. The fold change in EGFP expression for 4OHT (Snail+) cells was divided by EtOH (Snail−) cells for each compound to identify drugs that were capable of overcoming Snail-mediated EMT. To ensure the gain in EGFP expression was not simply a function of cell growth inhibition or cell death, we next compared the EGFP expression to the differential confluence in 4OHT-treated versus EtOH-treated cells. This analysis revealed a subset of compounds that led to differential re-activation of EGFP expression from the GIIIcI^2^ EMT/MET reporter while maintaining at least 50% viability or greater (Figure 3E). These included GSK2126458 (mTOR/PI3K), three microtubule associated agents, TAK-875 (GPR40 agonist), PIK-75 (DNA-PK, p110α), Sparfloxacin (antibiotic), LY2228820 (p38/MAPK), AUY922 (HSP90), and Edoxaban (Factor Xa) (Figures 3F, G).

### The chemical landscape of collateral sensitivity to enzalutamide-resistant prostate cancer

**FIGURE 4:**
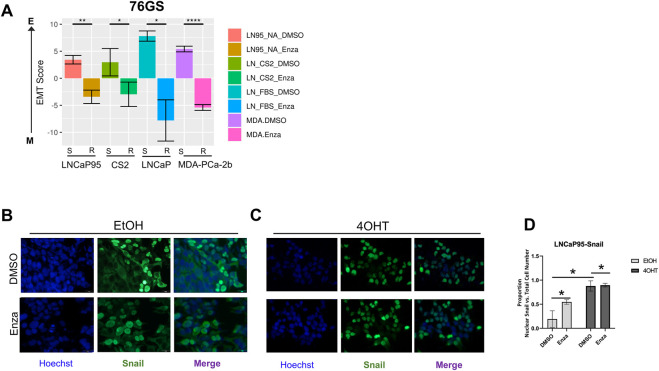
Enzalutamide induces epithelial plasticity. **(A)** Analysis of EMT scores across three isogenic pairs of independently-derived enzalutamide-sensitive and enzalutamide-resistant cell line models using the 76GS EMT scoring metric; s, enza-sensitive; r, enza-resistant. **(B)** Immunofluorescence staining of cell nuclei by Hoechst (blue) and Snail (green) in LNCaP95-Snail cells treated with EtOH (vehicle) and **(C)** 4OHT (nuclear Snail) in the presence of vehicle or enzalutamide. **(D)** Quantification of immunofluorescence by ImageJ. * = *p* < 0.05. All experiments were performed in triplicate.

Given the association between Snail-mediated EMT and enzalutamide resistance, we hypothesized that the evolution of enzalutamide resistance may also enrich for this EMT-like plasticity. To better understand these relationships between phenotypic plasticity and enzalutamide resistance we applied a series of EMT scoring metrics (Chakraborty et al., 2021; Subbalakshmi et al., 2021; Pillai et al., 2022) to analyze RNA-Seq data from four independent pairs of enzalutamide-sensitive and enzalutamide-resistant cell line models (Ware et al., 2020). Consistent with our hypothesis, enzalutamide-resistant cells exhibited a significant shift in scores toward a more mesenchymal-like gene expression signature (Figure 4A). These overall trends were consistent across scoring metrics, with some exceptions for specific cell line pairs, depending on the scoring metric used (Supplementary Figures S3A, B). Also consistent with this, treatment of LNCaP95-Snail(−) cells with enzalutamide led to an increase in nuclear localization of Snail (Figures 4B, D). The enzalutamide-treated LNCaP95-Snail cells mirrored induction of Snail nuclear localization with 4OHT treatment (Figures 4C, D). These analyses indicate that, compared to enzalutamide-sensitive cells, enzalutamide-resistant cells exhibit a more EMT-like phenotype.

**FIGURE 5:**
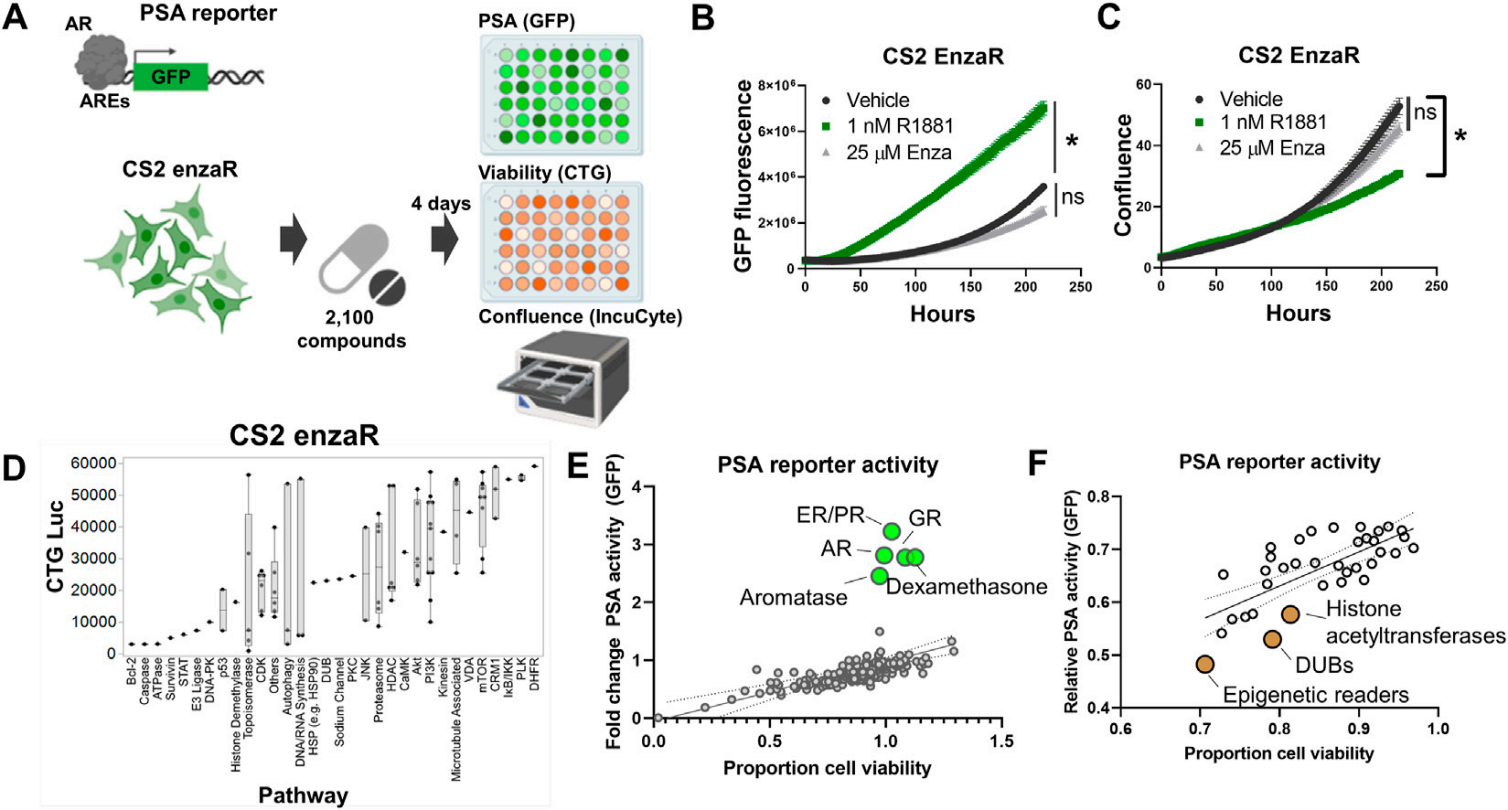
Collateral sensitivity screens identify candidate actionable pathways to treat enzalutamide-resistant prostate cancer. **(A)** PSA reporter schematic and screening strategy. **(B)** Validation of the PSA-GFP reporter system. **(C)** Confluence quantification in CS2 enzalutamide-resistant model following exposure to R1881 and enzalutamide. **(D)** Pathway-level analysis of top inhibitors targeting CS2 enzalutamide-resistant cells. **(E)** Activators of PSA reporter activity (green dots); top candidates are labeled by pathway. **(F)** Inhibitors of PSA reporter activity (brown dots); top candidates are labeled by pathway. All experiments were performed in triplicate.

To further extend the analysis of Snail-specific synthetic lethality, we next attempted to identify potential collateral sensitivities to this EMT-like enzalutamide-resistant phenotype. In order to accomplish this we performed a separate high-throughput compound screen on enzalutamide-resistant CS2 cells. The CS2 model is an LNCaP-derived subclone that was generated from long-term exposure to androgen deprivation through chronic culture in media containing charcoal-stripped fetal bovine serum (Ware et al., 2020). Subsequent exposure of enzalutamide-sensitive CS2 cells to increasing doses of enzalutamide over approximately 6 months led to the development of an enzalutamide-resistant CS2 cell line model (Ware et al., 2020). The CS2 enzalutamide-resistant model was transduced with a lentiviral PSA reporter in which the proximal promoter of PSA harboring androgen responsive elements is inserted upstream of the GFP reading frame (Figure 5A). These CS2^PSA−GFP^ enzalutamide-resistant cells were screened using the Bioactives library to interrogate AR signaling (GFP), ATP production (CellTiter Glo), and cell growth (IncuCyte imaging) (Figure 5A). To ensure the PSA reporter is responsive to androgen receptor signaling, cells were treated with the anabolic-androgenic steroid derivative, R1881, or enzalutamide. R1881 treatment led to a significant increase in GFP signal while enzalutamide had no effect on GFP expression in the enzalutamide-resistant CS2 model (Figure 5B). The increase in GFP during R1881 treatment was not due to a change in confluence, as these treatments did not increase cell confluence (Figure 5C). Analysis of cell growth inhibition for the Bioactives screen at the pathway level in the CS2 enzalutamide-resistant cells pinpointed candidate collateral sensitivities of interest, including DNA-PK, cyclin-dependent kinases, histone deacetylases, PI3K, mTOR, CRM1, and PLK (Figure 5D). Analysis of PSA reporter expression as a function of cell viability also revealed compounds targeting multiple receptors (androgen receptor, estrogen receptor, glucocorticoid receptor) as inducers of PSA reporter activity (Figure 5E) and compounds that target epigenetic modifiers as repressors of PSA reporter activity (Figure 5F).

**FIGURE 6:**
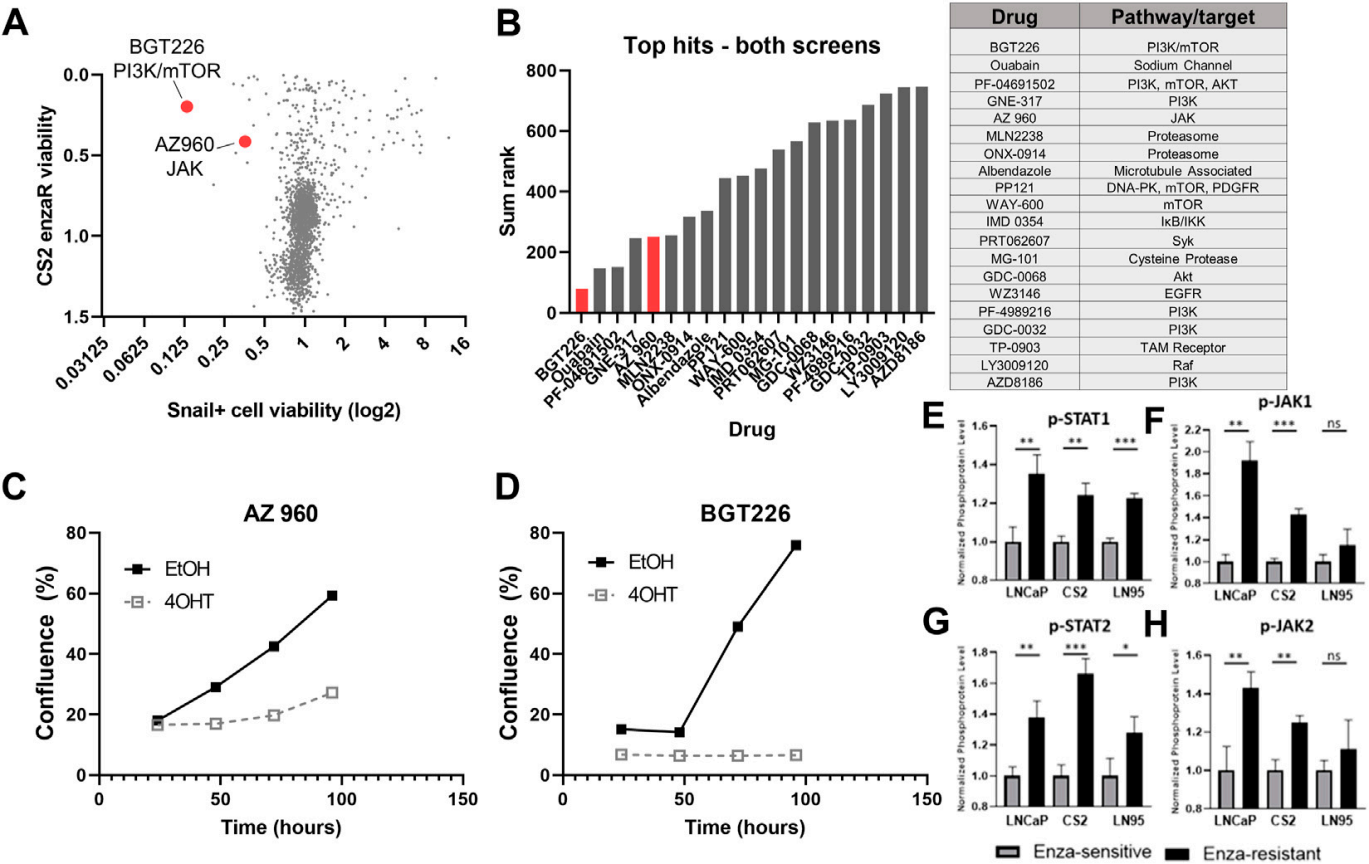
Comparison of candidate therapies for enzalutamide-resistant and Snail+ prostate cancer. **(A)** Comparison of CS2 enzaR and Snail drug screen hits. **(B)** Top hits for both screens based on a sum rank statistic that includes (CS2 enzaR confluence, Snail+ differential confluence, and Snail+ differential slope of growth rate). **(C)** Growth curves for LNCaP95 Snail EtOH (Snail−) and 4OHT (Snail+) cells treated with 1 μM AZ 960 (JAK inhibitor); and **(D)** BGT226 (PI3K/mTOR inhibitor). **(E)** Quantification of phospho-protein array data for p-STAT1, **(F)** p-JAK1, **(G)** p-STAT2, and **(H)** p-JAK2 in three pairs of enzalutamide-sensitive and enzalutamide-resistant models [Ware et al. (2020) biorxiv]. All experiments were performed in triplicate.

To provide further validation of candidates, we plotted the relative cell viability by CellTiter Glo for compounds in the CS2 enzaR screen by cell viability (CellTiter Glo) in the LNCaP95-Snail screen (Figure 6A). This analysis revealed a subset of drugs active in both screens. We ranked these top hits by a sum rank statistic that includes the rank of cell death by CellTiter for both screens as well as the differential confluence for Snail− vs. Snail+ cells (Figure 6B). Top targets from this analysis including PI3K, mTOR, and the proteasome (Figure 6B). Among this subset, AZ 960 (JAK2 inhibitor) and BGT226 (dual PI3K/mTOR inhibitor) were the most effective at inhibiting Snail+ cell confluence (Figures 6C, D). Differences in confluence are not likely due to changes in cell proliferation, as each value was normalized to the average confluence of untreated cells. These results were further validated in both LNCaP95-Snail and CS2 EnzaR cells with AZ 960 treatment using IC50 dose response curves (Supplementary Figure S4). Consistent with our observations of sensitivity to JAK2 inhibition, analysis of phospho-proteomics data from three previously-characterized pairs of enzalutamide-resistant lines (Ware et al., 2020), including CS2 enzalutamide-sensitive and -resistant lines demonstrates increased phosphorylation of STAT1, STAT2, JAK1, and JAK2 (Figures 6E–H), pinpointing the JAK/STAT signaling axis as a potential therapeutic vulnerability for Snail+ and enzalutamide-resistant prostate cancer.

## Discussion

In the present study we sought to characterize the therapeutic vulnerabilities for enzalutamide-resistant prostate cancer. To do this we combined high-throughput small molecule screens with real-time imaging and endpoint assays to reveal chemical landscapes of synthetic lethality for Snail-mediated EMT and collateral sensitivities for enzalutamide-resistant cells. These screens identified multiple therapeutic vulnerabilities of Snail+ prostate cancer cells, including several with known functions in prostate cancer and/or EMT, such as aurora kinases (Beltran et al., 2011; Kivinummi et al., 2017; Beltran et al., 2019), MET (Chu et al., 2014; Lucas et al., 2014), polo-like kinases (Weichert et al., 2004; Liu et al., 2011; Deeraksa et al., 2013), and CRM1/XPO1 (Gravina et al., 2017; Wei et al., 2018). The screen also pinpointed several inhibitors that differentially inhibited EMT as indicated by a change in the EMT reporter output without a change in confluence, including inhibitors of mTOR/PI3K, DNA-PK, and p38/MAPK (Figure 3H). All of these pathways have been previously connected to EMT biology in prostate cancer (Mulholland et al., 2012; Thakur et al., 2014; Zhang et al., 2019; Li et al., 2021). We also identified the GPR40 agonist, TAK-875, and Factor Xa inhibitor, Edoxoban, as potential inducers of MET. Consistent with these observations, another GPR40 agonist, GW9508, has been shown to prevent cytokine-induced airway epithelial barriers disruption of claudin, occludin, and ZO-1 (Moonwiriyakit et al., 2019), and Factor Xa inhibition has been shown to reduce EMT in chronic kidney disease (Fang et al., 2022). These agents represent promising candidates for follow-up studies to inhibit EMT and prevent or delay invasive and metastatic phenotypes associated with hormone therapy resistance.

Similar to the screen for Snail+ prostate cancer the follow-up screen for therapeutic vulnerabilities in enzalutamide-resistant CS2 cells pinpointed targets and pathways known to be involved in prostate cancer and hormone therapy resistance, including histone deacetylases, the PI3K/mTOR pathway, JAK-STAT signaling, DNA-PK, and Syk. For example, the identification of histone deacetylases and other epigenetic modifying agents is consistent with the known importance of epigenetic regulation of androgen receptor signaling (Rokhlin et al., 2006; Welsbie et al., 2009). Other targets, however, are linked to AR signaling bypass, as in the case of PTEN loss and subsequent constitutive activation of PI3K signaling (Carver et al., 2011), activation of JAK/STAT and FGFR signaling during the acquisition of AR independence and lineage plasticity (Chan et al., 2022; Deng et al., 2022), and the role of Syk as a potential mediator of invasive features and bone metastasis (Ghotra et al., 2015). While the relevance of these targets is well supported by preclinical evidence, the clinical utility of these targets is more varied. For example, our identification of mTOR/PI3K signaling inhibition as a key vulnerability may be the result of PTEN loss in these LNCaP-derived models (Lotan et al., 2011); however, while PTEN loss is also common among patients, these agents have been unsuccessful in clinical trials (Cham et al., 2021). Likewise, currently-available HDAC inhibitors have largely failed in clinical trials, mostly due to their toxicity (Bradley et al., 2009) or lack of efficacy (Molife et al., 2010; Eigl et al., 2015). Conversely, we also identified compounds targeting microtubules and microtubule dynamics in both screens. This is consistent with the use of taxane chemotherapy in the hormone therapy-resistant setting (Tannock et al., 2004). In terms of novel agents and pathways, there are a number of ongoing clinical trials for JAK inhibitors—particularly JAK2 inhibitors—in advanced prostate cancer, but thus far these have not demonstrated sufficient monotherapy activity in men with mCRPC [(Beinhoff et al., 2021) and see NCT00638378; closed due to lack of efficacy]. Our data suggest that a number of critical and non-redundant pathways may be involved in enzalutamide resistance and lineage plasticity, suggesting the need for combination trial approaches.

Comparison across both screens identified drugs with distinct effects in a single model as well as drugs that were common hits in both screens. There are multiple possible reasons for the observed differences in hits targeting each cell line, including, but not limited to, differences in the genetic and gene expression features of each cell line (Ware et al., 2020). For example, LNCaP95-Snail cells express AR-V7 while enzalutamide-resistant CS2 cells lack AR-V7. Enzalutamide-resistant CS2 cells also harbor dual loss of BRCA2 and RB1 and have a greater number of mutations and copy number alterations than LNCaP95 cells. These unique features may explain, at least in part, some of the differences in the list of hits from each screen.

A major limitation of the present study is the lack of *in vivo* modeling to validate the impact of our identified *in vitro* hits. This work is ongoing and also requires an assessment of the immune consequences of drug effects in the tumor microenvironment. Given the expression of mTOR, p38, and JAK/STAT signaling, for example, in immune cells and the immune suppressive impact of these agents in patients, assessing the net benefits of any drugs identified in our *in vitro* screen requires *in vivo* validation in a range of immunocompetent models either as monotherapy, in selected combinations and ideally in patient correlative samples.

The current study provides a platform to quantify the effects of thousands of compounds across multiple parameters and phenotypes simultaneously to identify and prioritize candidates for follow up in a rapid and cost-effective manner. While this study is limited by the exclusive use of *in vitro* cell line models, the integration of data from phenotypic reporters, confluence imaging, and CellTiter Glo readouts across multiple models rapidly identified a prioritized list of top hits, including the dual mTOR/PI3K inhibitor, BGT-226 and the JAK2 inhibitor, AZ-960, as promising candidates for future *in vivo* studies.

## Data Availability

The original contributions presented in the study are included in the article/Supplementary Material. Further inquiries can be directed to the corresponding authors.

## Supplementary material

SUPPLEMENTARY FIGURE S1
**(A)** Immunofluorescence staining of LNCaP95-Snail cells. EtOH serves as a vehicle for Snail induction. 4OHT induces localization of Snail and concomitant downregulation of E-cadherin and upregulation of vimentin.

SUPPLEMENTARY FIGURE S2
**(A)** Comparison of confluence for EtOH- and 4OHT-treated cells in untreated wells. **(B)** Correlation between CellTiter Glo and confluence. **(C)** Example of top drugs with differential growth slopes.

SUPPLEMENTARY FIGURE S3
**(A)** EMT scores for isogenic pairs of enzalutamide-sensitive and -resistant cell lines using the KS scoring metric and **(B)** the MLR scoring metric.

SUPPLEMENTARY FIGURE S4
**(A)** Growth curves for LNCaP95 Snail EtOH (Snail−) and 4OHT (Snail+) cells treated with 0.16 μM AZ 960 (JAK inhibitor). **(B)** Dose curve for LNCaP95 Snail EtOH (Snail−) and 4OHT (Snail+) cells treated with AZ 960 (JAK inhibitor) for 10 days. **(C)** Growth curves for CS2 EnzaR cells treated with 0.31 μM AZ 960 (JAK inhibitor). **(D)** Dose curve for CS2 EnzaR cells treated with AZ 960 (JAK inhibitor) for 10 days. *P < 0.05.

## References

[B1] Acar A. Nichol D. Fernandez-Mateos J. Cresswell G. D. Barozzi I. Hong S. P. (2020). Exploiting evolutionary steering to induce collateral drug sensitivity in cancer. Nat. Commun. 11 (1), 1923. 10.1038/s41467-020-15596-z 32317663 PMC7174377

[B2] Altunel E. Roghani R. S. Chen K. Y. Kim S. Y. McCall S. Ware K. E. (2020). Development of a precision medicine pipeline to identify personalized treatments for colorectal cancer. BMC Cancer 20 (1), 592. 10.1186/s12885-020-07090-y 32580713 PMC7313200

[B3] Alumkal J. J. Sun D. Lu E. Beer T. M. Thomas G. V. Latour E. (2020). Transcriptional profiling identifies an androgen receptor activity-low, stemness program associated with enzalutamide resistance. Proc. Natl. Acad. Sci. U. S. A. 117 (22), 12315–12323. 10.1073/pnas.1922207117 32424106 PMC7275746

[B4] Armstrong A. J. Azad A. A. Iguchi T. Szmulewitz R. Z. Petrylak D. P. Holzbeierlein J. (2022). Improved survival with enzalutamide in patients with metastatic hormone-sensitive prostate cancer. J. Clin. Oncol. 40 (15), 1616–1622. 10.1200/JCO.22.00193 35420921 PMC9113211

[B5] Bai Y. Zhang Z. Cheng L. Wang R. Chen X. Kong Y. (2019). Inhibition of enhancer of zeste homolog 2 (EZH2) overcomes enzalutamide resistance in castration-resistant prostate cancer. J. Biol. Chem. 294 (25), 9911–9923. 10.1074/jbc.RA119.008152 31085587 PMC6597805

[B6] Beer T. M. Armstrong A. J. Rathkopf D. E. Loriot Y. Sternberg C. N. Higano C. S. (2014). Enzalutamide in metastatic prostate cancer before chemotherapy. N. Engl. J. Med. 371 (5), 424–433. 10.1056/NEJMoa1405095 24881730 PMC4418931

[B7] Beinhoff P. Sabharwal L. Udhane V. Maranto C. LaViolette P. S. Jacobsohn K. M. (2021)., 13. Basel), 5204. 10.3390/cancers13205204 Second-generation Jak2 inhibitors for advanced prostate cancer: Are we ready for clinical development? Cancers 20 34680353 PMC8533841

[B8] Beltran H. Oromendia C. Danila D. C. Montgomery B. Hoimes C. Szmulewitz R. Z. (2019). A phase II trial of the aurora kinase A inhibitor alisertib for patients with castration-resistant and neuroendocrine prostate cancer: Efficacy and biomarkers. Clin. Cancer Res. 25 (1), 43–51. 10.1158/1078-0432.CCR-18-1912 30232224 PMC6320304

[B9] Beltran H. Rickman D. S. Park K. Chae S. S. Sboner A. MacDonald T. Y. (2011). Molecular characterization of neuroendocrine prostate cancer and identification of new drug targets. Cancer Discov. 1 (6), 487–495. 10.1158/2159-8290.CD-11-0130 22389870 PMC3290518

[B10] Blatt E. B. Raj G. V. (2019). Molecular mechanisms of enzalutamide resistance in prostate cancer. Cancer Drug Resist 2 (2), 189–197. 10.20517/cdr.2019.25 35582713 PMC8992629

[B11] Bradley D. Rathkopf D. Dunn R. Stadler W. M. Liu G. Smith D. C. (2009). Vorinostat in advanced prostate cancer patients progressing on prior chemotherapy (national cancer institute trial 6862): Trial results and interleukin-6 analysis: A study by the department of defense prostate cancer clinical trial consortium and university of chicago phase 2 consortium. Cancer 115 (23), 5541–5549. 10.1002/cncr.24597 19711464 PMC2917101

[B12] Byers L. A. Diao L. Wang J. Saintigny P. Girard L. Peyton M. (2013). An epithelial-mesenchymal transition gene signature predicts resistance to EGFR and PI3K inhibitors and identifies Axl as a therapeutic target for overcoming EGFR inhibitor resistance. Clin. Cancer Res. 19 (1), 279–290. 10.1158/1078-0432.CCR-12-1558 23091115 PMC3567921

[B13] Carver B. S. Chapinski C. Wongvipat J. Hieronymus H. Chen Y. Chandarlapaty S. (2011). Reciprocal feedback regulation of PI3K and androgen receptor signaling in PTEN-deficient prostate cancer. Cancer Cell 19 (5), 575–586. 10.1016/j.ccr.2011.04.008 21575859 PMC3142785

[B14] Chakraborty P. Chen E. L. McMullen I. Armstrong A. J. Kumar Jolly M. Somarelli J. A. (2021). Analysis of immune subtypes across the epithelial-mesenchymal plasticity spectrum. Comput. Struct. Biotechnol. J. 19, 3842–3851. 10.1016/j.csbj.2021.06.023 34306571 PMC8283019

[B15] Cham J. Venkateswaran A. R. Bhangoo M. (2021). Targeting the PI3K-AKT-mTOR pathway in castration resistant prostate cancer: A review article. Clin. Genitourin. Cancer 19 (6), 563 e1–e563563.e7. 10.1016/j.clgc.2021.07.014 34433523

[B16] Chan J. M. Zaidi S. Love J. R. Zhao J. L. Setty M. Wadosky K. M. (2022). Lineage plasticity in prostate cancer depends on JAK/STAT inflammatory signaling. Science 377 (6611), 1180–1191. 10.1126/science.abn0478 35981096 PMC9653178

[B17] Chi K. N. Agarwal N. Bjartell A. Chung B. H. Pereira de Santana Gomes A. J. Given R. (2019). Apalutamide for metastatic, castration-sensitive prostate cancer. N. Engl. J. Med. 381 (1), 13–24. 10.1056/NEJMoa1903307 31150574

[B18] Chu G. C. Zhau H. E. Wang R. Rogatko A. Feng X. Zayzafoon M. (2014). RANK- and c-Met-mediated signal network promotes prostate cancer metastatic colonization. Endocr. Relat. Cancer 21 (2), 311–326. 10.1530/ERC-13-0548 24478054 PMC3959765

[B19] Cutti L. Rigon C. A. G. Kaspary T. E. Turra G. M. Markus C. Merotto A. (2021). Negative cross-resistance to clomazone in imazethapyr-resistant Echinochloa crus-galli caused by increased metabolization. Pestic. Biochem. Physiol. 178, 104918. 10.1016/j.pestbp.2021.104918 34446194

[B20] Deeraksa A. Pan J. Sha Y. Liu X. D. Eissa N. T. Lin S. H. (2013). Plk1 is upregulated in androgen-insensitive prostate cancer cells and its inhibition leads to necroptosis. Oncogene 32 (24), 2973–2983. 10.1038/onc.2012.309 22890325 PMC3499666

[B21] Deng S. Wang C. Wang Y. Xu Y. Li X. Johnson N. A. (2022). Ectopic JAK-STAT activation enables the transition to a stem-like and multilineage state conferring AR-targeted therapy resistance. Nat. Cancer 3 (9), 1071–1087. 10.1038/s43018-022-00431-9 36065066 PMC9499870

[B22] Eigl B. J. North S. Winquist E. Finch D. Wood L. Sridhar S. S. (2015). A phase II study of the HDAC inhibitor SB939 in patients with castration resistant prostate cancer: NCIC clinical trials group study IND195. Invest. New Drugs 33 (4), 969–976. 10.1007/s10637-015-0252-4 25983041

[B23] Fang L. Ohashi K. Ogawa H. Otaka N. Kawanishi H. Takikawa T. (2022). Factor Xa inhibitor, edoxaban ameliorates renal injury after subtotal nephrectomy by reducing epithelial-mesenchymal transition and inflammatory response. Physiol. Rep. 10 (5), e15218. 10.14814/phy2.15218 35262272 PMC8905573

[B24] Fizazi K. Shore N. Tammela T. L. Ulys A. Vjaters E. Polyakov S. (2019). Darolutamide in nonmetastatic, castration-resistant prostate cancer. N. Engl. J. Med. 380 (13), 1235–1246. 10.1056/NEJMoa1815671 30763142

[B25] Fizazi K. Tran N. Fein L. Matsubara N. Rodriguez-Antolin A. Alekseev B. Y. (2017). Abiraterone plus prednisone in metastatic, castration-sensitive prostate cancer. N. Engl. J. Med. 377 (4), 352–360. 10.1056/NEJMoa1704174 28578607

[B26] Formaggio N. Rubin M. A. Theurillat J. P. (2021). Loss and revival of androgen receptor signaling in advanced prostate cancer. Oncogene 40 (7), 1205–1216. 10.1038/s41388-020-01598-0 33420371 PMC7892335

[B27] George J. T. Jolly M. K. Xu S. Somarelli J. A. Levine H. (2017). Survival outcomes in cancer patients predicted by a partial EMT gene expression scoring metric. Cancer Res. 77 (22), 6415–6428. 10.1158/0008-5472.CAN-16-3521 28947416 PMC5690883

[B28] Ghotra V. P. He S. van der Horst G. Nijhoff S. de Bont H. Lekkerkerker A. (2015). SYK is a candidate kinase target for the treatment of advanced prostate cancer. Cancer Res. 75 (1), 230–240. 10.1158/0008-5472.CAN-14-0629 25388286

[B29] Gravina G. L. Mancini A. Colapietro A. Marampon F. Sferra R. Pompili S. (2017). Pharmacological treatment with inhibitors of nuclear export enhances the antitumor activity of docetaxel in human prostate cancer. Oncotarget 8 (67), 111225–111245. 10.18632/oncotarget.22760 29340049 PMC5762317

[B30] Hu R. Lu C. Mostaghel E. A. Yegnasubramanian S. Gurel M. Tannahill C. (2012). Distinct transcriptional programs mediated by the ligand-dependent full-length androgen receptor and its splice variants in castration-resistant prostate cancer. Cancer Res. 72 (14), 3457–3462. 10.1158/0008-5472.CAN-11-3892 22710436 PMC3415705

[B31] Huggins C. Hodges C. V. (1972). Studies on prostatic cancer. I. The effect of castration, of estrogen and androgen injection on serum phosphatases in metastatic carcinoma of the prostate. CA Cancer J. Clin. 22 (4), 232–240. 10.3322/canjclin.22.4.232 4625049

[B32] Imamovic L. Sommer M. O. (2013). Use of collateral sensitivity networks to design drug cycling protocols that avoid resistance development. Sci. Transl. Med. 5 (204), 204ra132. 10.1126/scitranslmed.3006609 24068739

[B33] James N. D. de Bono J. S. Spears M. R. Clarke N. W. Mason M. D. Dearnaley D. P. (2017). Abiraterone for prostate cancer not previously treated with hormone therapy. N. Engl. J. Med. 377 (4), 338–351. 10.1056/NEJMoa1702900 28578639 PMC5533216

[B34] Jindal R. Nanda A. Pillai M. Ware K. E. Singh D. Sehgal M. (2023). Emergent dynamics of underlying regulatory network links EMT and androgen receptor-dependent resistance in prostate cancer. Comput. Struct. Biotechnol. J. 21, 1498–1509. 10.1016/j.csbj.2023.01.031 36851919 PMC9957767

[B35] Kirkman L. A. Zhan W. Visone J. Dziedziech A. Singh P. K. Fan H. (2018). Antimalarial proteasome inhibitor reveals collateral sensitivity from intersubunit interactions and fitness cost of resistance. Proc. Natl. Acad. Sci. U. S. A. 115 (29), E6863–E6870. 10.1073/pnas.1806109115 29967165 PMC6055138

[B36] Kivinummi K. Urbanucci A. Leinonen K. Tammela T. L. J. Annala M. Isaacs W. B. (2017). The expression of AURKA is androgen regulated in castration-resistant prostate cancer. Sci. Rep. 7 (1), 17978. 10.1038/s41598-017-18210-3 29269934 PMC5740165

[B37] Ku S. Y. Rosario S. Wang Y. Mu P. Seshadri M. Goodrich Z. W. (2017). Rb1 and Trp53 cooperate to suppress prostate cancer lineage plasticity, metastasis, and antiandrogen resistance. Science 355 (6320), 78–83. 10.1126/science.aah4199 28059767 PMC5367887

[B38] Labrecque M. P. Alumkal J. J. Coleman I. M. Nelson P. S. Morrissey C. (2021). The heterogeneity of prostate cancers lacking AR activity will require diverse treatment approaches. Endocr. Relat. Cancer 28 (8), T51–T66. 10.1530/ERC-21-0002 33792558 PMC8292199

[B39] Li Q. Deng Q. Chao H. P. Liu X. Lu Y. (2018). Linking prostate cancer cell AR heterogeneity to distinct castration and enzalutamide responses. Nat. Commun. 9 (1), 3600. 10.1038/s41467-018-06067-7 30190514 PMC6127155

[B40] Li S. Sheng J. Liu Z. Fan Y. Zhang C. Lv T. (2021). Potent antitumour of the mTORC1/2 dual inhibitor AZD2014 in docetaxel-sensitive and docetaxel-resistant castration-resistant prostate cancer cells. J. Cell Mol. Med. 25 (5), 2436–2449. 10.1111/jcmm.16155 33507584 PMC7933970

[B41] Liu X. S. Song B. Elzey B. D. Ratliff T. L. Konieczny S. F. Cheng L. (2011). Polo-like kinase 1 facilitates loss of Pten tumor suppressor-induced prostate cancer formation. J. Biol. Chem. 286 (41), 35795–35800. 10.1074/jbc.C111.269050 21890624 PMC3195584

[B42] Lotan T. L. Gurel B. Sutcliffe S. Esopi D. Liu W. Xu J. (2011). PTEN protein loss by immunostaining: Analytic validation and prognostic indicator for a high risk surgical cohort of prostate cancer patients. Clin. Cancer Res. 17 (20), 6563–6573. 10.1158/1078-0432.CCR-11-1244 21878536 PMC3195839

[B43] Lucas J. M. Heinlein C. Kim T. Hernandez S. A. Malik M. S. True L. D. (2014). The androgen-regulated protease TMPRSS2 activates a proteolytic cascade involving components of the tumor microenvironment and promotes prostate cancer metastasis. Cancer Discov. 4 (11), 1310–1325. 10.1158/2159-8290.CD-13-1010 25122198 PMC4409786

[B44] Miao L. Yang L. Li R. Rodrigues D. N. Crespo M. Hsieh J. T. (2017). Disrupting androgen receptor signaling induces snail-mediated epithelial-mesenchymal plasticity in prostate cancer. Cancer Res. 77 (11), 3101–3112. 10.1158/0008-5472.CAN-16-2169 28302679

[B45] Molife L. R. Attard G. Fong P. C. Karavasilis V. Reid A. H. M. Patterson S. (2010). Phase II, two-stage, single-arm trial of the histone deacetylase inhibitor (HDACi) romidepsin in metastatic castration-resistant prostate cancer (CRPC). Ann. Oncol. 21 (1), 109–113. 10.1093/annonc/mdp270 19608618

[B46] Moonwiriyakit A. Koval M. Muanprasat C. (2019). Pharmacological stimulation of G-protein coupled receptor 40 alleviates cytokine-induced epithelial barrier disruption in airway epithelial Calu-3 cells. Int. Immunopharmacol. 73, 353–361. 10.1016/j.intimp.2019.05.026 31129422 PMC6620115

[B47] Mu P. Zhang Z. Benelli M. Karthaus W. R. Hoover E. Chen C. C. (2017). SOX2 promotes lineage plasticity and antiandrogen resistance in TP53- and RB1-deficient prostate cancer. Science 355 (6320), 84–88. 10.1126/science.aah4307 28059768 PMC5247742

[B48] Mulholland D. J. Kobayashi N. Ruscetti M. Zhi A. Tran L. M. Huang J. (2012). Pten loss and RAS/MAPK activation cooperate to promote EMT and metastasis initiated from prostate cancer stem/progenitor cells. Cancer Res. 72 (7), 1878–1889. 10.1158/0008-5472.CAN-11-3132 22350410 PMC3319847

[B49] Pillai M. Rajaram G. Thakur P. Agarwal N. Muralidharan S. Ray A. (2022). Mapping phenotypic heterogeneity in melanoma onto the epithelial-hybrid-mesenchymal axis. Front. Oncol. 12, 913803. 10.3389/fonc.2022.913803 36003764 PMC9395132

[B50] Rao S. R. Somarelli J. A. Altunel E. Selmic L. E. Byrum M. Sheth M. U. (2020). From the clinic to the bench and back again in one dog year: How a cross-species pipeline to identify new treatments for sarcoma illuminates the path forward in precision medicine. Front. Oncol. 10, 117. 10.3389/fonc.2020.00117 32117764 PMC7026496

[B51] Rodriguez Y. Unno K. Truica M. I. Chalmers Z. R. Yoo Y. A. Vatapalli R. (2022). A genome-wide CRISPR activation screen identifies PRRX2 as a regulator of enzalutamide resistance in prostate cancer. Cancer Res. 82 (11), 2110–2123. 10.1158/0008-5472.CAN-21-3565 35405009 PMC9177667

[B52] Rokhlin O. W. Glover R. B. Guseva N. V. Taghiyev A. F. Kohlgraf K. G. Cohen M. B. (2006). Mechanisms of cell death induced by histone deacetylase inhibitors in androgen receptor-positive prostate cancer cells. Mol. Cancer Res. 4 (2), 113–123. 10.1158/1541-7786.MCR-05-0085 16513842

[B53] Scher H. I. Fizazi K. Saad F. Taplin M. E. Sternberg C. N. Miller K. (2012). Increased survival with enzalutamide in prostate cancer after chemotherapy. N. Engl. J. Med. 367 (13), 1187–1197. 10.1056/NEJMoa1207506 22894553

[B54] Schroeder A. Herrmann A. Cherryholmes G. Kowolik C. Buettner R. Pal S. (2014). Loss of androgen receptor expression promotes a stem-like cell phenotype in prostate cancer through STAT3 signaling. Cancer Res. 74 (4), 1227–1237. 10.1158/0008-5472.CAN-13-0594 24177177 PMC4539262

[B55] Smith M. R. Saad F. Chowdhury S. Oudard S. Hadaschik B. A. Graff J. N. (2018). Apalutamide treatment and metastasis-free survival in prostate cancer. N. Engl. J. Med. 378 (15), 1408–1418. 10.1056/NEJMoa1715546 29420164

[B56] Somarelli J. A. Armstrong A. J. Sheth M. U. Ware K. E. (2020). “Phenotypic plasticity and lineage switching in prostate cancer,” in Phenotypic switching. Editor Levine H. (United states: Academic Press), 591–615.

[B57] Somarelli J. A. DeGregori J. Gerlinger M. Heng H. H. Marusyk A. Welch D. R. (2022). Questions to guide cancer evolution as a framework for furthering progress in cancer research and sustainable patient outcomes. Med. Oncol. 39 (9), 137. 10.1007/s12032-022-01721-z 35781581 PMC9252949

[B58] Somarelli J. A. Roghani R. S. Moghaddam A. S. Thomas B. C. Rupprecht G. Ware K. E. (2020). A precision medicine drug discovery pipeline identifies combined CDK2 and 9 inhibition as a novel therapeutic strategy in colorectal cancer. Mol. Cancer Ther. 19 (12), 2516–2527. 10.1158/1535-7163.MCT-20-0454 33158998 PMC7718319

[B59] Somarelli J. A. Rupprecht G. Altunel E. Flamant E. M. Rao S. Sivaraj D. (2020). A comparative Oncology drug discovery pipeline to identify and validate new treatments for osteosarcoma. Cancers (Basel) 12 (11), 3335. 10.3390/cancers12113335 33187254 PMC7696249

[B60] Somarelli J. A. Schaeffer D. Bosma R. Bonano V. I. Sohn J. W. Kemeny G. (2013). Fluorescence-based alternative splicing reporters for the study of epithelial plasticity *in vivo* . RNA 19 (1), 116–127. 10.1261/rna.035097.112 23185039 PMC3527723

[B61] Somarelli J. A. Schaeffer D. Marengo M. S. Bepler T. Rouse D. Ware K. E. (2016). Distinct routes to metastasis: Plasticity-dependent and plasticity-independent pathways. Oncogene 35 (33), 4302–4311. 10.1038/onc.2015.497 26751776 PMC4940344

[B62] Somarelli J. A. (2021). The hallmarks of cancer as ecologically driven phenotypes. Front. Ecol. Evol. 9, 661583. 10.3389/fevo.2021.661583 34703824 PMC8544241

[B63] Stoyanova T. Cooper A. R. Drake J. M. Liu X. Armstrong A. J. Pienta K. J. (2013). Prostate cancer originating in basal cells progresses to adenocarcinoma propagated by luminal-like cells. Proc. Natl. Acad. Sci. U. S. A. 110 (50), 20111–20116. 10.1073/pnas.1320565110 24282295 PMC3864278

[B64] Subbalakshmi A. R. Sahoo S. McMullen I. Saxena A. N. Venugopal S. K. Somarelli J. A. (2021). KLF4 induces mesenchymal-epithelial transition (MET) by suppressing multiple EMT-inducing transcription factors. Cancers (Basel) 13 (20), 5135. 10.3390/cancers13205135 34680284 PMC8533753

[B65] Tan T. Z. Miow Q. H. Miki Y. Noda T. Mori S. Huang R. Y. J. (2014). Epithelial-mesenchymal transition spectrum quantification and its efficacy in deciphering survival and drug responses of cancer patients. EMBO Mol. Med. 6 (10), 1279–1293. 10.15252/emmm.201404208 25214461 PMC4287932

[B66] Tannock I. F. de Wit R. Berry W. R. Horti J. Pluzanska A. Chi K. N. (2004). Docetaxel plus prednisone or mitoxantrone plus prednisone for advanced prostate cancer. N. Engl. J. Med. 351 (15), 1502–1512. 10.1056/NEJMoa040720 15470213

[B67] Thakur N. Gudey S. K. Marcusson A. Fu J. Y. Bergh A. Heldin C. H. (2014). TGFβ-induced invasion of prostate cancer cells is promoted by c-Jun-dependent transcriptional activation of Snail1. Cell Cycle 13 (15), 2400–2414. 10.4161/cc.29339 25483191 PMC4128885

[B68] Vega S. Morales A. V. Ocaña O. H. Valdés F. Fabregat I. Nieto M. A. (2004). Snail blocks the cell cycle and confers resistance to cell death. Genes Dev. 18 (10), 1131–1143. 10.1101/gad.294104 15155580 PMC415638

[B69] Ware K. E. Gupta S. Eng J. Kemeny G. Puviindran B. J. Foo W. C. (2020). Convergent evolution of p38/MAPK activation in hormone resistant prostate cancer mediates pro-survival, immune evasive, and metastatic phenotypes. bioRxiv. 2020.

[B70] Ware K. E. Somarelli J. A. Schaeffer D. Li J. Zhang T. Park S. (2016). Snail promotes resistance to enzalutamide through regulation of androgen receptor activity in prostate cancer. Oncotarget 7 (31), 50507–50521. 10.18632/oncotarget.10476 27409172 PMC5226599

[B71] Wazir S. Shad S. A. (2022). Development of fipronil resistance, fitness cost, cross-resistance to other insecticides, stability, and risk assessment in Oxycarenus hyalinipennis (Costa). Sci. Total Environ. 803, 150026. 10.1016/j.scitotenv.2021.150026 34500277

[B72] Wei X. X. Siegel A. P. Aggarwal R. Lin A. M. Friedlander T. W. Fong L. (2018). A phase II trial of selinexor, an oral selective inhibitor of nuclear export compound, in abiraterone- and/or enzalutamide-refractory metastatic castration-resistant prostate cancer. Oncologist 23 (6), 656–e64. 10.1634/theoncologist.2017-0624 29487219 PMC6067936

[B73] Weichert W. Schmidt M. Gekeler V. Denkert C. Stephan C. Jung K. (2004). Polo-like kinase 1 is overexpressed in prostate cancer and linked to higher tumor grades. Prostate 60 (3), 240–245. 10.1002/pros.20050 15176053

[B74] Welsbie D. S. Xu J. Chen Y. Borsu L. Scher H. I. Rosen N. (2009). Histone deacetylases are required for androgen receptor function in hormone-sensitive and castrate-resistant prostate cancer. Cancer Res. 69 (3), 958–966. 10.1158/0008-5472.CAN-08-2216 19176386 PMC3219545

[B75] Zhang G. Gurtu V. Kain S. R. (1996). An enhanced green fluorescent protein allows sensitive detection of gene transfer in mammalian cells. Biochem. Biophys. Res. Commun. 227 (3), 707–711. 10.1006/bbrc.1996.1573 8885998

[B76] Zhang J. Jiang H. Xu D. Wu W. J. Chen H. D. (2019). DNA-PKcs mediates an epithelial-mesenchymal transition process promoting cutaneous squamous cell carcinoma invasion and metastasis by targeting the TGF-β1/smad signaling pathway. Onco Targets Ther. 12, 9395–9405. 10.2147/OTT.S205017 31807020 PMC6844265

