# Supplementary figures and images for: A synthetic lethal screen for Snail-induced enzalutamide resistance identifies JAK/STAT signaling as a therapeutic vulnerability in prostate cancer
Source: Front Mol Biosci. 2023 May 9;10:1104505. doi: 10.3389/fmolb.2023.1104505 (PMC10203420; doi:10.3389/fmolb.2023.1104505)

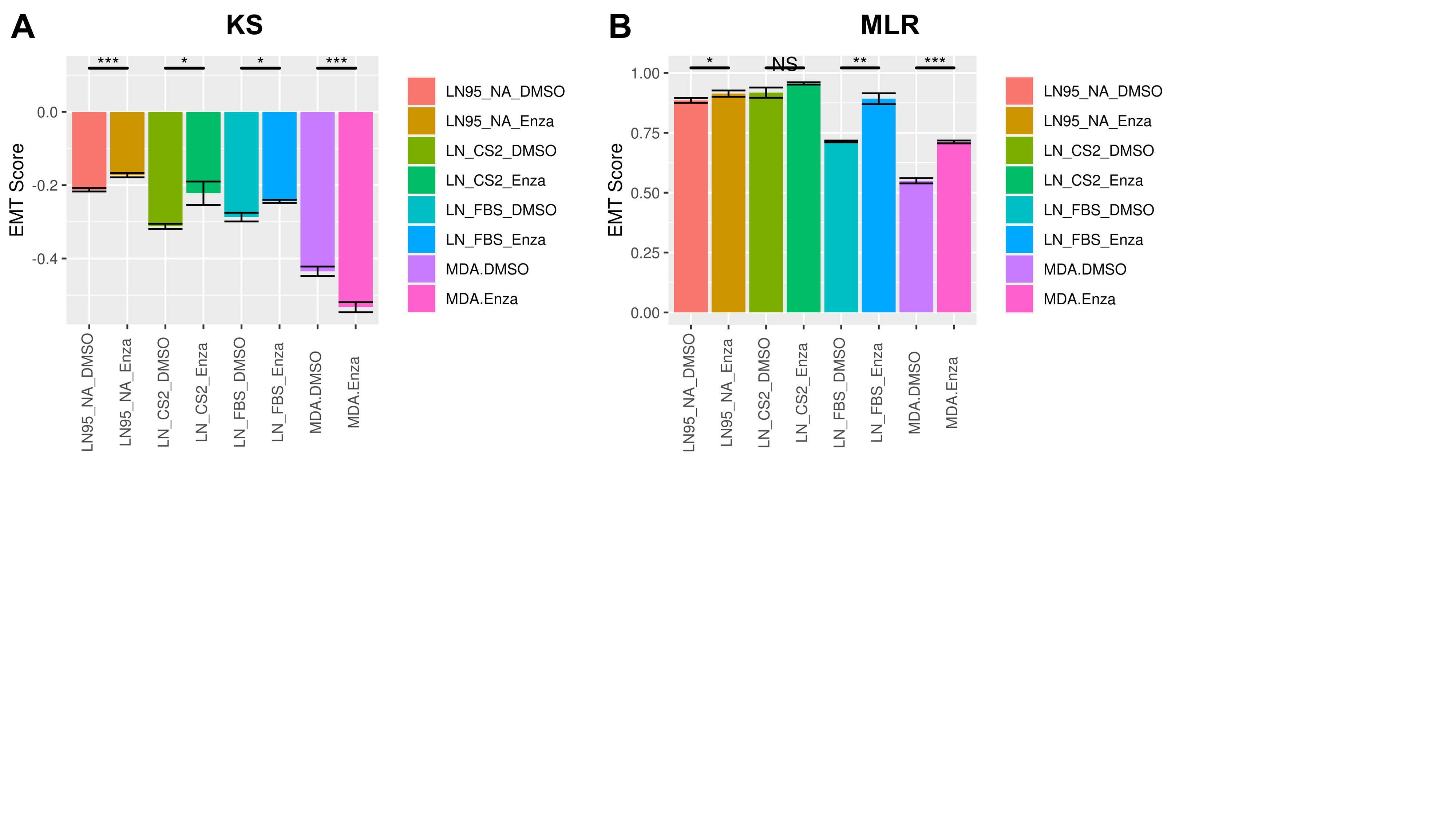

Supplement: Supplementary file 1 [file Image3.TIF]

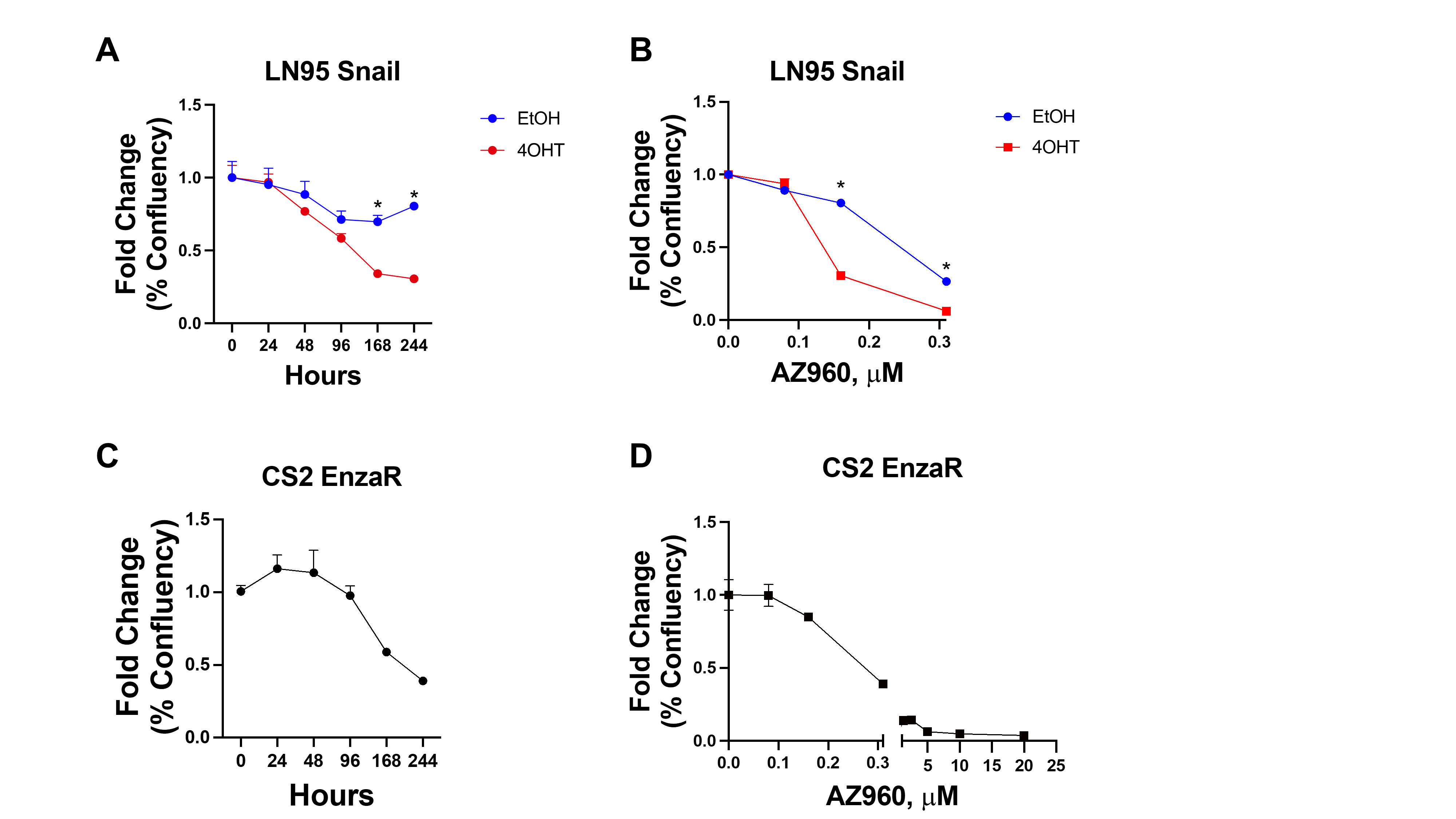

Supplement: Supplementary file 2 [file Image4.TIF]

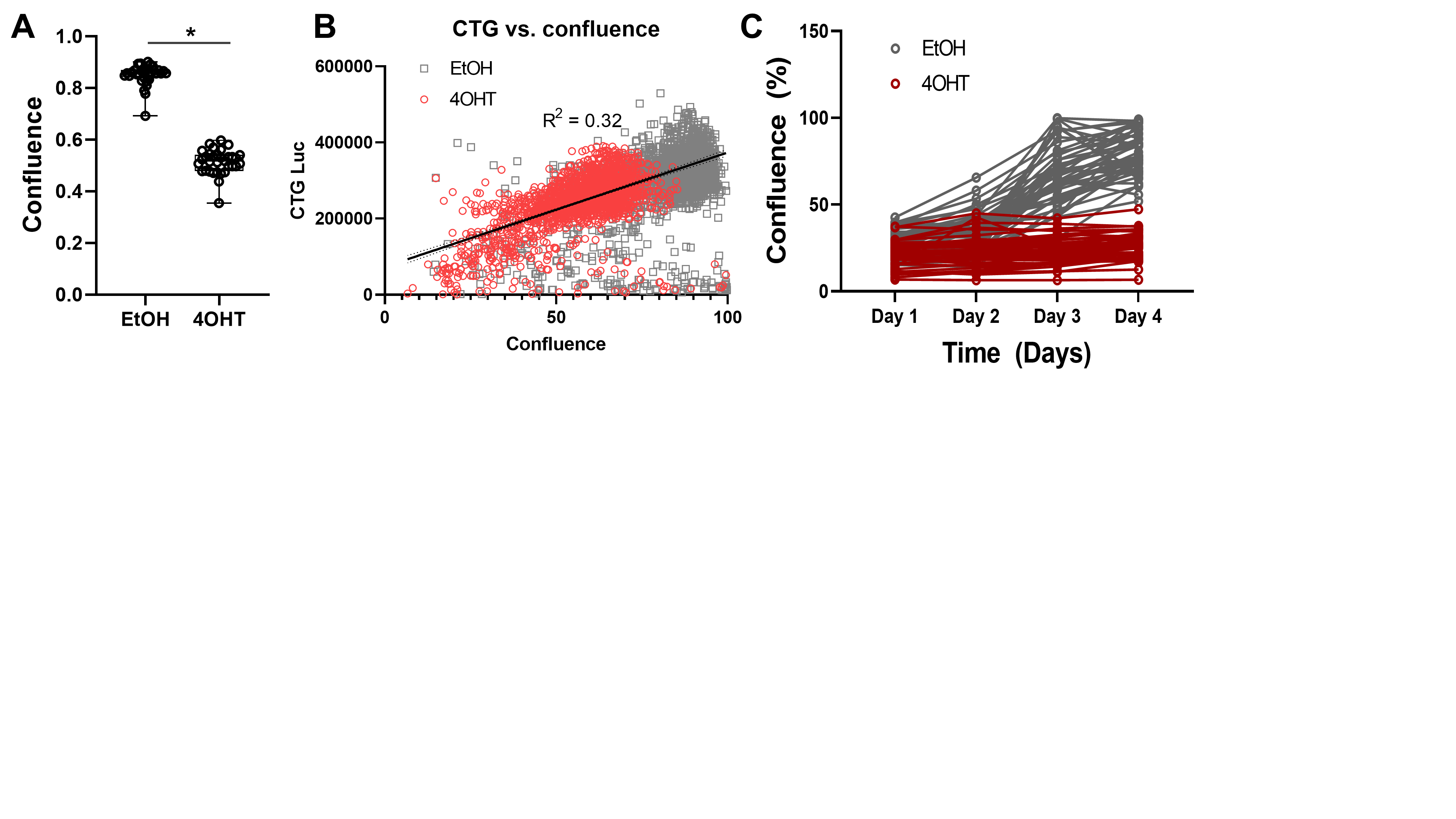

Supplement: Supplementary file 3 [file Image2.TIF]

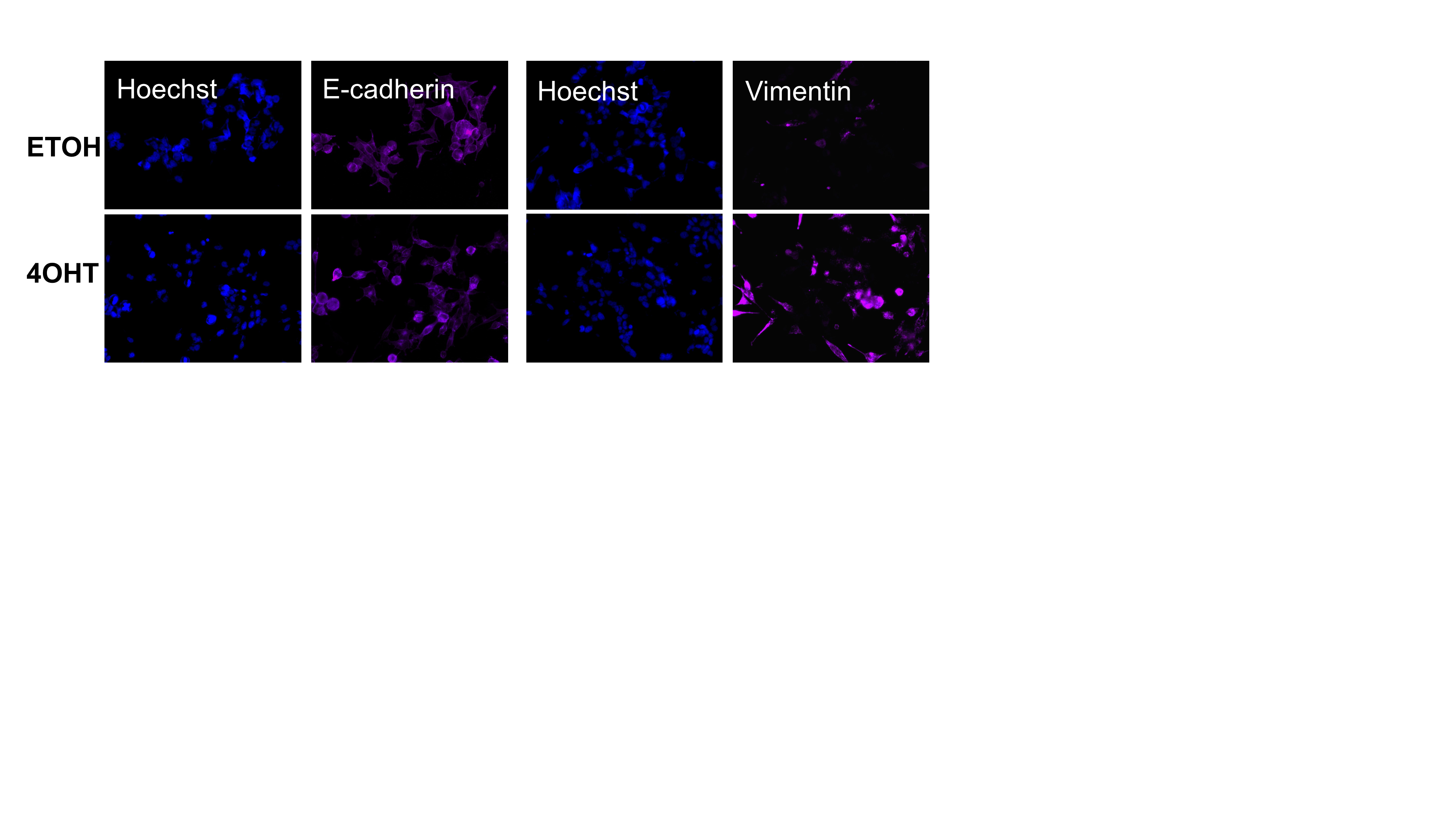

Supplement: Supplementary file 4 [file Image1.TIF]
